# Hemodialysis Catheter-Associated Right Atrial Thrombus Diagnosed via Point of Care Transesophageal Echocardiogram

**DOI:** 10.24908/pocus.v9i1.16895

**Published:** 2024-04-22

**Authors:** Heather Andrade, Julie Carroll, Evan Tomkiewicz, Edwin Jackson

**Affiliations:** 1 Internal Medicine-Pediatrics Residency Program, Indiana University School of Medicine Indianapolis, IN USA; 2 Division of Pulmonary and Critical Care Medicine, Indiana University School of Medicine Indianapolis, IN USA

**Keywords:** Thrombus, TEE, Catheter Associated Right Atrial Thrombus

## Abstract

Catheter-associated right atrial thrombus (CRAT) is a potential complication of central venous catheter placement and is associated with an increase in morbidity and mortality. The precise incidence of CRAT is unknown, and there is a lack of clear screening and management guidelines for this condition. Additionally, the diagnosis is often missed when using transthoracic echocardiography (TTE) alone. Here, we present a case of a 64-year-old female admitted to the medical intensive care unit with multiorgan dysfunction who was diagnosed with hemodialysis catheter-associated right atrial thrombus (HDCRAT) via intensivist-performed point of care transesophageal echocardiography (TEE) after an initial TTE was negative. This patient was successfully treated with systemic anticoagulation, local thrombolysis, and delayed removal of the temporary hemodialysis catheter. Our experience serves to highlight the improved visualization of the right atrium and the diagnostic superiority of HDCRAT with TEE. We suspect that with greater utilization of TEE among intensivists, CRAT and HDCRAT will have increased recognition. It is imperative that intensivists are aware of this complication and various management strategies. Still, more studies are needed to establish clear management guidelines for CRAT and the associated complications.

## Introduction

Catheter-associated right atrial thrombus (CRAT) is a cause of significant morbidity in adult and pediatric patients following the placement of central venous catheters (CVCs). Potential complications of CRAT include pulmonary embolism, infection, septic emboli, arrhythmia, tricuspid regurgitation, catheter malfunction, superior vena cava obstruction, and in cases of CRAT associated with hemodialysis (HD) (hemodialysis catheter-associated right atrial thrombus (HDCRAT)), the loss of vascular access in the affected vein and incomplete dialysis 1, 2. In a retrospective study of published cases of HDCRAT in the adult HD population before 2010, mortality was reported at 18.3% 2. Thus, early identification and treatment of HDCRAT is crucial. The pathogenesis of HDCRAT primarily arises from the mechanical irritation of the right atrium (RA) by the movement of the catheter tip with cardiac contraction. This persistent irritation leads to endothelial injury, platelet aggregation, and activation of the coagulation cascade, culminating in the development of a thrombus. Notwithstanding the associated risk of HDCRAT, the Kidney Disease Outcomes Quality Initiative (KDOQI) endorses the placement of the HD catheter tip in the RA, emphasizing that this approach facilitates greater blood flow rates, thereby enhancing dialysis efficiency 3, 4, 5.

Although CRAT and HDCRAT are well-known complications of CVC placement, the true incidence has yet to be accurately determined due to limitations in imaging the RA 6. In the classical approach to identifying masses within the RA, the diagnosis is established through transesophageal echocardiography (TEE). This preference arises from the inherent challenges when using transthoracic echocardiography (TTE) to closely visualize the RA and catheter tip 6, 7, 8. Retrospective studies of diagnosed CRAT cases have shown an incidence of roughly 5%. However, autopsy reports have estimated the incidence to be closer to 30% 6. Therefore, it is speculated that many cases of CRAT remain clinically undetected until complications arise, which raises further concerns about the inadequacy of TTE.

When imaging the RA, TEE outperforms TTE primarily because of its multiplane imaging capabilities, which enable a comprehensive 180-degree visualization of cardiac structures 6, 7, 8. However, TTE is more frequently utilized than TEE because of its availability and familiarity by intensivists. Unfortunately, estimates show that TTE may miss as many as 50% of right atrial masses 7, 8, 9. Given the limitations in imaging right atrial thrombi with TTE, physicians have been unable to make this diagnosis at the bedside, leading to delays in identification and treatment 5, 6, 7.

Recently, the increasing prevalence of point of care ultrasound (POCUS) TEE within medical intensive care units (ICUs) has afforded intensivists the ability to more readily identify and diagnose CRATs in the ICU setting 10. Here, we present a case of a 64-year-old female who was diagnosed with HDCRAT via intensivist-performed TEE following an comprehensive TTE.

## Case Presentation

**Figure 1:**
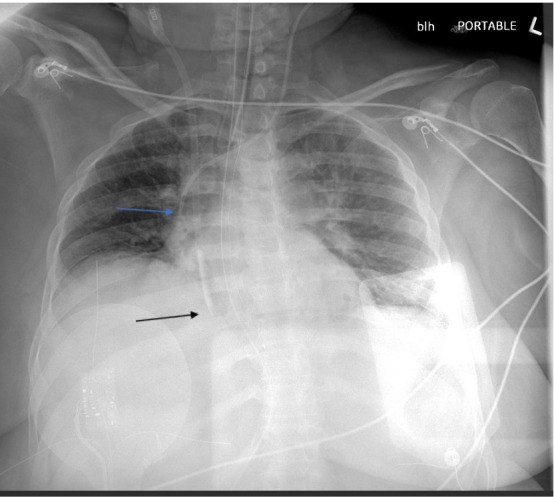
12Fr R IJ HD catheter terminating in the mid-RA (black arrow). 7 Fr L IJ CVC terminating in the caval atrial junction (blue arrow)

A 64-year-old African American female presented to the ICU with multi-organ dysfunction (including acute kidney injury (AKI), hypoxic respiratory failure, circulatory failure, and severe metabolic acidosis) due to metformin toxicity and aspiration pneumonia. She was initially intubated and subsequently treated with broad-spectrum antimicrobials. She required norepinephrine, vasopressin, and epinephrine to maintain a mean arterial pressure of 65 mmHg. A temporary right internal jugular (IJ) dialysis catheter was placed terminating in the mid-RA, and a left IJ CVC was inserted with the catheter tip positioned at the caval-atrial junction (Figure 1). She started continuous renal replacement therapy (CRRT) for the treatment of severe acidosis and AKI. 

**Figure 2:**
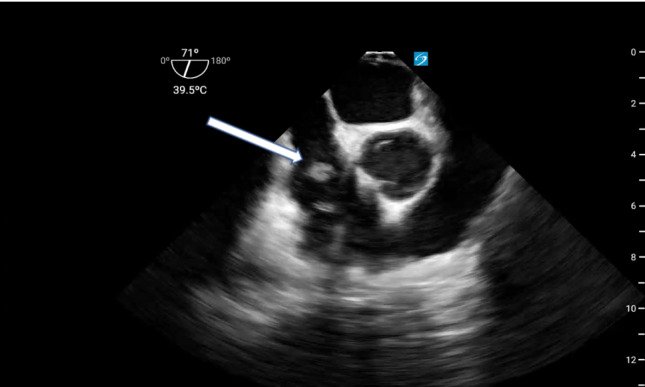
MERV inflow outflow view showing the CRAT (white arrow).

**Figure 3:**
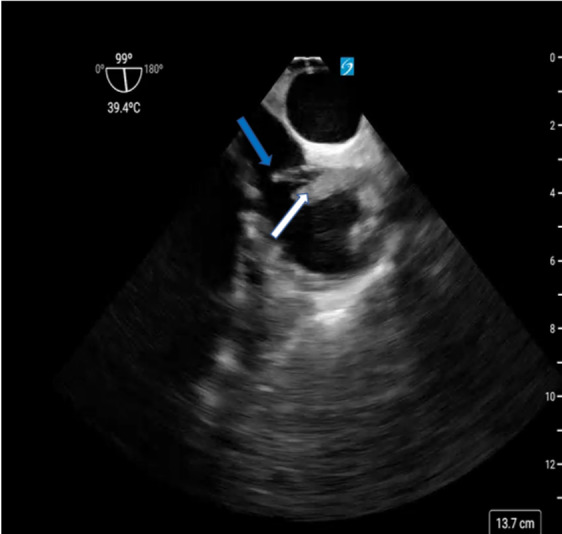
Modified ME bi-caval view showing the CRAT (white arrow) and the HD catheter (blue arrow).

**Figure 4:**
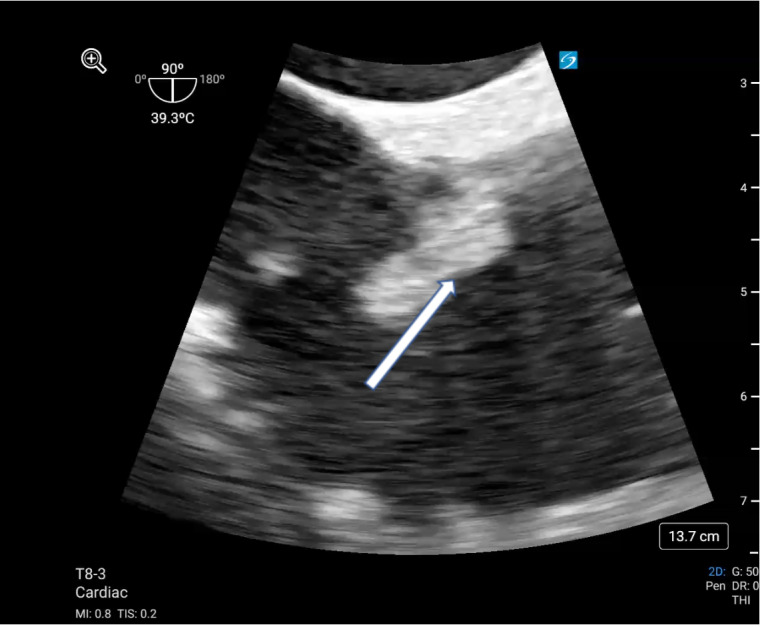
Zoomed MEbicaval view showing the CRAT in the RA (white arrow).

Despite improvement of her metabolic derangements, she remained profoundly hypotensive and hypoxic in the setting of three-vasopressor shock. On the second day of CRRT, several clots were extracted from the distal port of the right IJ dialysis catheter. A transthoracic echocardiogram (TTE) of good quality was performed and revealed clear visualization of all left ventricular walls as well as adequate views of the mitral, tricuspid, and aortic valves. This assessment revealed hyperdynamic function of both the right and left ventricles, absence of valvular pathology or pericardial effusion, and a left ventricular outflow tract velocity time integral measured at 26.6 cm. Notably, the right IJ HD catheter was not observed in the RA on the TTE. Following our institutional protocol, a non-diagnostic TTE in a patient with undifferentiated shock necessitates further investigation with a POCUS TEE. Consequently, we performed a POCUS TEE that acquired standard Critical Care TEE views, including midesophageal (ME) four chamber, ME bi-caval, ME long axis, and transgastric short axis views. This evaluation excluded cardiac tamponade, dysfunction in both the left and right ventricles, and hypovolemia as potential causes of her shock. Specifically, the normal function of the right ventricle suggested that an acute pulmonary embolism was an unlikely shock cause. Adjustment of the multi-plane to 71 degrees within the mid-esophagus produced the ME right ventricle inflow/outflow view, revealing the presence of a mobile density within the RA (Figure 2 & video S1). Subsequent adjustment of the multi-plane to 99 degrees created the ME bicaval view, allowing for the visualization and measurement of a 1.8 cm right atrial thrombus affixed to the right IJ dialysis catheter (Figure 2,3&4).

Given the POCUS TEE findings, therapeutic anticoagulation was immediately initiated with intravenous heparin. Due to the clot's location and size, removal of the catheter was deferred because of a high risk of clot embolization. Instead, therapeutic anticoagulation was continued, and tissue plasminogen activator (tPA) locking solution was introduced into the catheter. This combined approach aimed to concentrate the tPA at the catheter tip, effectively serving as a local regional thrombolytic therapy. Subsequently, the patient experienced an improvement in renal function and clinical status, leading to her extubation, discontinuation of vasopressors and CRRT. She remained on anticoagulation for one week, and the right IJ HD catheter was successfully removed without complications. While her clinical improvement was likely not directly attributable to the diagnosis and treatment of HDCRAT, the therapeutic intervention played a crucial role in preventing further clot propagation and reducing the risk of pulmonary embolism in this critically ill patient. Consequently, given her positive clinical trajectory, a decision was made not to perform a repeat POCUS TEE.

## Discussion

CRAT is often insidious and a potentially life-threatening complication that may arise in association with any CVC. However, it predominantly presents in cases involving tunneled HD catheters, typically emerging 12 weeks post-catheter insertion 1. There is a paucity of data describing the occurrence of HDCRAT in the context of temporary HD catheters used for acute renal replacement therapy in critically ill medical ICU patients. The practice of positioning HD catheters within the RA is formally recommended by the KDOQI guidelines and is a recognized risk factor for HDCRAT development 2. In this paper, we presented a case of HDCRAT originating from a temporary dialysis catheter, which eluded detection through TTE and was incidentally diagnosed through intensivist-performed TEE.

The precise incidence of CRAT remains elusive. Reports from clinical studies have presented a wide range, varying from 18% to 30%, accompanied by mortality rates spanning 9% to 18% 4, 5, 6. In its clinical presentation, CRAT often manifests as catheter dysfunction stemming from mechanical obstruction by the thrombus, as in this case. Two predominant forms of CRAT are recognized: mural thrombus and catheter tip thrombus. Both forms share a common etiology, induced by mechanical trauma to the atrial wall caused by the catheter, coupled with myocardial contractions leading to endothelial damage and activation of the clotting cascade. This ultimately culminates in catheter dysfunction and CRAT. 

Compared to TTE, TEE has consistently demonstrated its diagnostic superiority with enhanced resolution, multiplane imaging capabilities, and improved visualization of the RA and its contents 11, 12, 13. In the past decade, there has been a noticeable paradigm shift in the application of TEE 14, 15, 16. This shift has extended its utility beyond cardiologists and cardiothoracic anesthesiologists, to employment by intensivists at the bedside 1. Notably, Lau et al. have substantiated that intensivist-performed limited TEE exhibits diagnostic accuracy equal to that achieved by cardiologists 15. Given the increasing prominence of TEE, it is paramount for intensivists to maintain an acute awareness of CRAT and to acquaint themselves with the array of management strategies at their disposal. 

Current management guidelines for HDCRAT in patients undergoing HD and those in the ICU lack clarity. Expert consensus and insights gleaned from clinical case reports favor a multifaceted approach, primarily revolving around systemic anticoagulation and subsequent catheter removal. This approach is further supplemented by the judicious use of antibiotic prophylaxis in less severe cases 6, 12. In instances where CRAT features substantial thrombi exceeding 6 cm in length or accompanied by cardiac abnormalities or endocarditis, the consensus recommends a more aggressive therapeutic approach entailing surgical thrombectomy 11. 

Within the specific context of our experience, a nuanced, hybrid strategy was employed. This approach combined the use of anticoagulation with localized thrombolytic therapy – a protocol initially described by Gilon et al. 12. The decision to employ this hybrid approach was contingent upon the unique clinical intricacies of the patient's condition, offering a tailored solution to optimize management. The evolving landscape of HDCRAT management warrants continual assessment and adaptation to develop a more consistent patient-centered approach. The development of more refined evidence-based guidelines is essential to enhance the management of HDCRAT in critically ill patients. Further research and collaborative efforts are required to determine the most effective and safe treatment strategies.

## Conclusion

We suspect that the detection of HDCRAT cases will increase due to the use of intensivist-performed TEE, allowing for better bedside right atrial imaging. With this increase in use, intensivists should be aware of this diagnosis and possible management strategies. Furthermore, additional investigations are warranted to assess whether the benefits of enhanced high blood-flow rates and increased efficiency of dialysis, attributed to the placement of HD catheter tips in the RA, truly outweigh the accompanying risks. 

## Conflicts of Interest

None

## Consent

Written consent was obtained from the patient for publication of this case report including images.

## Supplementary Material

 Video S1 ME RV inflow outflow view showing the CRAT.
